# Are Image-Based Deep Learning Algorithms of Kidney Volume in Polycystic Kidney Disease Ready for Clinical Deployment? A Systematic Review and Meta-Analysis

**DOI:** 10.3390/jcm14228255

**Published:** 2025-11-20

**Authors:** Emil Colliander, Sebastian Tupper, Mira Lansner Kielberg, Marie Louise Liu, Enrique Almar-Munoz, Agnes Mayr, Rebeca Mirón Mombiela

**Affiliations:** 1 Department of Radiology, Herlev and Gentofte Hospital, 2730 Herlev, Denmark; emil.colliander@regionh.dk (E.C.); sebastian.tupper@regionh.dk (S.T.); mira.lansner.kielberg@regionh.dk (M.L.K.); marie.louise.en-ting.liu.02@regionh.dk (M.L.L.); 2 Department of Radiology, Medical University of Innsbruck, 6020 Innsbruck, Austria; enrique.almar@i-med.ac.at (E.A.-M.); a.mayr@i-med.ac.at (A.M.); 3 Institute for Clinical Medicine, University of Copenhagen, 2200 Copenhagen, Denmark

**Keywords:** autosomal dominant polycystic kidney disease (ADPKD), total kidney volume (TKV), deep learning, artificial intelligence (AI), diagnostic imaging, CT, MRI, ultrasound, systematic review, meta-analysis

## Abstract

**Objectives:** In patients with autosomal dominant polycystic kidney disease (ADPKD), total kidney volume (TKV) is the gold standard biomarker for assessing the risk of progression and the need for drug therapy. However, it is a time-consuming process. In this systematic review and meta-analysis, we evaluate the current state of deep learning (DL) algorithms for automatic kidney volume segmentation. **Methods:** All original research, including the search terms ADPKD, diagnostic imaging, DL, and TKV, was identified in PubMed, Embase, and Ovid MEDLINE databases from January 2000 to 13 October 2024. Articles with insufficient information to assess methodological quality were excluded. Quality was assessed using the “Quality Assessment of Diagnostic Accuracy Studies, Version 2” (QUADAS-2) and Checklist for Artificial Intelligence in Medical Imaging (CLAIM) tools. We focused on the Dice Similarity Coefficient (DSC), bias differences, and time efficiency as outcomes. **Results:** Nineteen studies were included, with an overall low risk of bias; however, the mean adherence to the CLAIM checklist was 64%. The pooled DSC under the random-effects model was 0.953 (95% CI: 0.938–0.969) with relatively low bias for TKV in 5622 ADPKD patients (mean age, 46.1 years; 45% male) and 9180 scans (79% MRI). The average segmentation time was decreased by 75% compared to the ground truth. Performance differences were evident among imaging modalities, MRI sequences, and 3D vs. 2D models, but not among imaging planes. The between-study heterogeneity was low ($I^{2}=0\%$), and no statistically significant evidence of small-study effects or publication bias was detected. **Conclusions:** DL models for TKV in ADPKD patients demonstrated high precision compared to manual segmentation in a large, pooled sample with heterogeneous study designs and methods. While clinical implementation is not yet feasible, the current work demonstrates the technical and diagnostic efficacy of image-based DL segmentation models.

## 1. Introduction

Autosomal dominant polycystic kidney disease (ADPKD) is the most common genetic kidney disorder globally, with an incidence of approximately 1 in every 400 to 1000 live births [1,2]. Most cases are caused by PKD1 and PKD2 locus mutations, accounting for approximately 78% and 15%, respectively [3].

ADPKD is characterized by the progressive development and expansion of bilateral fluid-filled cysts, distortion of the renal parenchyma, and increased kidney volumes [4]. This leads to progressive loss of renal function, measured by the estimated glomerular filtration rate (eGFR), and accounts for 5–10% of end-stage renal disease (ESRD) cases [4,5]. The eGFR is a poor predictor of future decline in renal function, as it is often normal until the late stages of the disease.

Increased cyst burden and kidney volume precede the loss of renal function. Therefore, the total kidney volume (TKV) adjusted for height and age was introduced to predict future decline in eGFR [6]. This is relevant due to the emerging role of vasopressin receptor antagonists in inhibiting cyst growth and renal functional decline, which is reserved for moderate- to high-risk patients according to the Mayo Clinic Imaging Classification due to the high cost of treatment [6]. For these reasons, medical imaging plays a crucial role in risk assessment and treatment management in patients with ADPKD.

TKV increases gradually by approximately 2–5% per year [7,8], indicating a need for reproducible and operator-independent methods to measure TKV accurately. Manual segmentation is the most accurate measurement method. However, manually tracing the renal outline on cross-sectional imaging is time-consuming, with studies reporting analysis times ranging from 15 to 45 min per kidney [9]. To facilitate and reduce reporting times, the application of deep learning algorithms for automated kidney segmentation and TKV measurement in ADPKD is emerging. Several studies have developed or tested deep learning (DL) models for TKV and cyst segmentation; however, an overview of the general feasibility of these algorithms is not yet available. Therefore, this systematic review and meta-analysis will summarize and synthesize current data on image-based DL models for TKV measurement in ADPKD patients and analyze the factors that affect their generalization ability and clinical deployment. The research objective is to examine kidney volume measurements by using image-based DL segmentation models in patients with ADPKD and compare them to ground truth (GT) defined by manual segmentation or semi-automatic segmentation performed by imaging experts.

## 2. Materials and Methods

Ethical approval was not required for this systematic review. The protocol is registered on the PROSPERO International Prospective Register of Systematic Reviews (CRD42024611910) and was reported according to the Preferred Reporting Items for Systematic Reviews and Meta-Analyses (PRISMA) of Diagnostic Test Accuracy Studies Checklist [10,11]. The current article checklist is available in Table A1.

### 2.1. Search Strategy

We searched the PubMed, EMBASE, and Ovid MEDLINE databases to identify suitable studies. Studies published from 1 January 2000 to 13 October 2024 were included in the analysis. Filters for human participants and English-worded studies were applied. The search strategies and syntax used to search the databases are available in Appendix B. Additional studies that are cited in other systematic reviews during record screening or in the included studies and that fulfilled the eligibility criteria of this systematic review were included. An information specialist from Copenhagen University Library, Health/Science, was consulted during the process.

### 2.2. Study Selection

The PICO (Patient/Population, Intervention, Comparison, Outcome) framework was used to structure the reporting of eligibility criteria. Studies were included if (1) they encompassed patients with ADPKD in humans of all ages and sexes; (2) imaging included computed tomography (CT), magnetic resonance imaging (MRI), and/or ultrasonography (US) techniques and protocols; (3) they used DL models with segmentation architectures; and (4) they reported TKV measurements. Non-original research (case reports, reviews, commentary, letters to the editor), preprints, conference papers, and short communications were excluded.

After the initial search of the electronic databases, the abstracts and metadata were transferred to rayyan.ai, a systematic review software [12]. After eliminating duplicates, two reviewers screened titles and abstracts independently to determine eligibility. Screening of the full text of publications was performed if the abstracts required additional information to determine eligibility. All included studies needed to be approved by at least two authors (M.L.K. and S.T.). Uncertainties were resolved through consensus of a third author (R.M.).

### 2.3. Assessment of Study Quality and Risk of Bias

The quality of the selected studies was rigorously assessed using the Checklist for Artificial Intelligence in Medical Imaging (CLAIM) [13] alongside the Quality Assessment of Diagnostic Accuracy Studies, Version 2 (QUADAS-2) [14]. The QUADAS-2 tool was modified in two ways. First, the risk of bias was assessed as either low, some concerns, high, or unclear. Second, even though the QUADAS was developed for the assessment of diagnostic accuracy, precision, and/or repeatability, studies are affected by similar biases as diagnostic accuracy studies; therefore, the tool was still applied to the DL segmentation models, as precision evaluation is a common denominator in these studies. Both tools were used in all studies, with four reviewers (M.L.K., S.T., E.C., and M.L.L.) undertaking the primary appraisal. All reviewers worked independently, but in instances of discrepancy, the senior investigator (R.M.M.) was consulted to ensure a consensus-based, comprehensive evaluation. It is essential to clarify that while the CLAIM checklist is not a tool for determining risk of bias, it did serve as a best-practice tool to ensure that the reporting of AI research was comprehensive and consistent, thereby enabling the potential for clinical translation.

### 2.4. Data Extraction

Qualified articles were read thoroughly, and relevant data were extracted, including the first author and year of publication, publication type, study design, country, funding source and corresponding author contact information, data source, number of patients, number of scans, age (*mean and standard deviation (SD)/range*), sex, modality, imaging protocols and acquisition, ground truth, dataset sizes, data stratification (training, validation, etc.), segmentation methodology, Dice Similarity Coefficient (DSC), mean absolute error, Blant–Altman analysis, limitations, time-efficiency analysis, and data code-sharing policies. No assumptions were made about any missing data or unclear information; instead, it was simply recorded as ‘*not available*’ or ‘*unclear*’.

### 2.5. Data Synthesis and Statistical Analysis

We summarized and analyzed data from high-quality literature and calculated combined discrimination statistics. For the segmentation task, we conducted a meta-analysis of studies that offered summary statistics of the DSC with confidence intervals from out-of-sample external validations, considering both geographically and temporally validated data. We focused on the highest-scoring algorithm in a study that presented multiple DSCs, as varying algorithms were employed. The meta-analysis was conducted using Python 3.12 with the statsmodels.stats.meta_analysis module (version 0.14.0), employing the DerSimonian–Laird random-effects model to estimate the pooled DSC. The primary-effect measure was the mean DSC, with variance calculated as the squared standard deviation of each study’s reported DSC. Studies lacking dispersion metrics (e.g., standard deviations or confidence intervals) were excluded from the quantitative synthesis, and the number of excluded cases was explicitly reported. Subgroup analysis and meta-regressions were used to explore sources of variability, such as modality (US vs. CT vs. MRI), MRI sequences (T1, T2, STIR, etc.), or DL segmentation techniques (2D vs. 3D models). No other variables affecting the volume measurement were suspected or pre-specified at this time. Sensitivity analyses were performed to evaluate the influence of low-performing studies and confirm the robustness of the pooled estimate. To evaluate inter-study heterogeneity, visual inspection of forest plots was used, Q and I^2^ statistics were calculated to quantify the percentage of variation due to true differences, and a 95% prediction interval was also calculated. Publication bias was assessed with a funnel plot and Egger’s publication bias test.

## 3. Results

### 3.1. Study Selection

**Figure 1:**
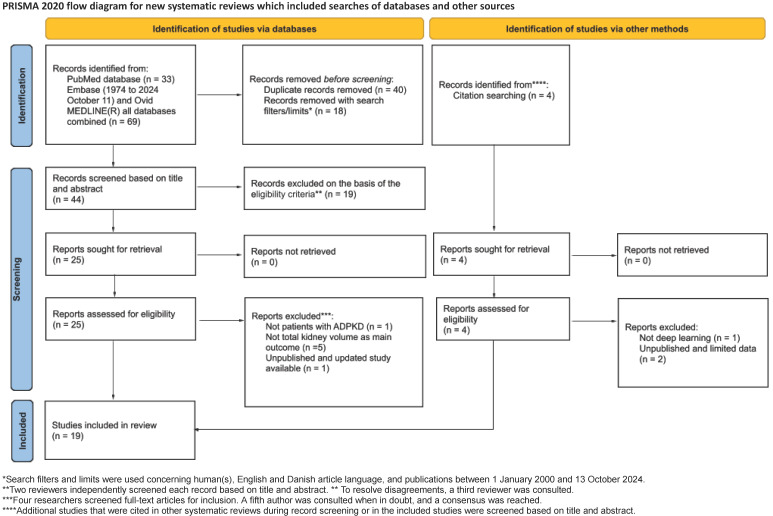
Preferred Reporting Items for Systematic Reviews and Meta-Analyses flowchart of the article screening and selection process.

The PRISMA flow diagram (Figure 1) shows that the database search yielded 44 unique results after excluding duplicates and applying the search filters. Nineteen studies were excluded during the title and abstract screening, leaving 25 to undergo full-text screening. These studies were assessed for eligibility through a full-text review, and 15 were included in the systematic review. Four articles were identified through citations, resulting in a total of 19 studies included in the systematic review. Reasons for exclusion during the full-text screening are listed in the PRISMA flow diagram.

Of the studies identified for full-text screening, 10 that appeared to meet the inclusion criteria were excluded upon review for various reasons. One investigated how a neural network, U-Net, is used to evaluate visceral fat [15]; one reported the wrong outcome (cyst measurement instead of TKV) [16]; one was a preprint, with no peer review identified and a vaguely defined ground truth [17]; one focused on predictors of renal volume rather than the artificial intelligence (AI) methods used to assess TKV [18]; one focused on the implementation of attention maps to achieve better DL performance [19]; one reported TKV, but it was not height-adjusted as in the other studies [20]; one studied the wrong condition (patients with kidney and liver tumors rather than ADPKD patients) [21]; one used methods that were not deep learning-based [22]; and one compared several DL methods [23]. The last one was a conference paper that lacked the necessary details to be included [24].

### 3.2. Systematic Review Characteristics

Table 1 presents the characteristics of the 19 included studies conducted between 2017 and 2024; patient cohorts ranged from 18 to 2173 across Asia, Europe, and North America, encompassing a total of 5622 patients with ADPKD and 9180 scans. The patients’ mean age was 46.1 years, ranging from 16 to 74 years, with an estimated 45% of patients being male; however, the sex of the patients was underreported in the included studies. Most studies employed a retrospective study design, but one study was prospective [25], and seven studies conducted prospective testing [26,27,28,29,30,31,32]. Studies varied in structure, encompassing single-center (11%) and multi-center settings (89%), with a primary focus on MRI (79%), although some studies also used CT (16%) or US (5%) as imaging modalities. Dataset segmentation into training and testing sets was prevalent, with manual validation consistently used, emphasizing the importance of human intervention in model validation. However, 21% of the studies used semi-automatic segmentation. Almost two-thirds (58%) of the segmentations were performed by a radiologist, while the remaining segmentations were conducted by a nephrologist, clinical expert, or investigator supervised by a radiologist. The DL models spanned classical encoder–decoder designs, such as 2D/3D U-Nets (68%) and V-Nets (5%) with tailored loss weighting, to more advanced attention-augmented and transformer-based models (11%), as well as ensemble strategies that integrate 2D and 3D feature representations (5%). Several groups further leveraged efficient encoders (16%) and multimodal inputs to enhance cross-sequence generalization (31.58%). Most studies reported whether they had conflicts of interest; only two studies did not [29,33].

Even though one of our predetermined outcomes was the time efficiency of the DL models, this parameter was highly underreported. Only 37% of studies calculated this data, and the results were reported heterogeneously. One study reported the rate in slices/hour [34], while others reported the rate in minutes per scan [26,29,35,36]. Additionally, two studies compared the rates for the right versus left kidney [27,31]. To make the metrics comparable, we reported the findings in Shin et al. using minutes per image slice. Overall, we determined a significant time reduction between the manual and semi-automatic segmentation methods versus the DL models, with an average decrease of 75%, ranging from 42% to 99.8%, based on the provided data (Table 1). Because the data from Sharma et al. [35] were estimates, we omitted this data from the calculation.

### 3.3. Meta-Analysis of the Factors That Affect the Generalization Ability of the DL Models

Table 2 presents the Dice scores and biases in the TKV (%) for all included studies, along with factors that may affect the study metrics. The DSC values ranged from 79.5% to 98%, with an average of 94.74 ± 2.89% (95% CI: 94.62–94.85). We calculated the pooled DSC across studies by weighting each study’s reported mean DSC by the number of scans in its test set. To quantify variability, we calculated the weighted standard deviation and derived the standard error of the pooled mean, again taking into account the number of scans in each study. The percentage bias in TKV differences was calculated for 17 of the 19 studies, as data were missing for the remaining two. Most studies (79%) revealed minor differences, while 12% showed no significant differences between the standard segmentation method and the DL models. Of those that showed differences, 47% overestimated the volume and 31% underestimated it.

**Figure 2:**
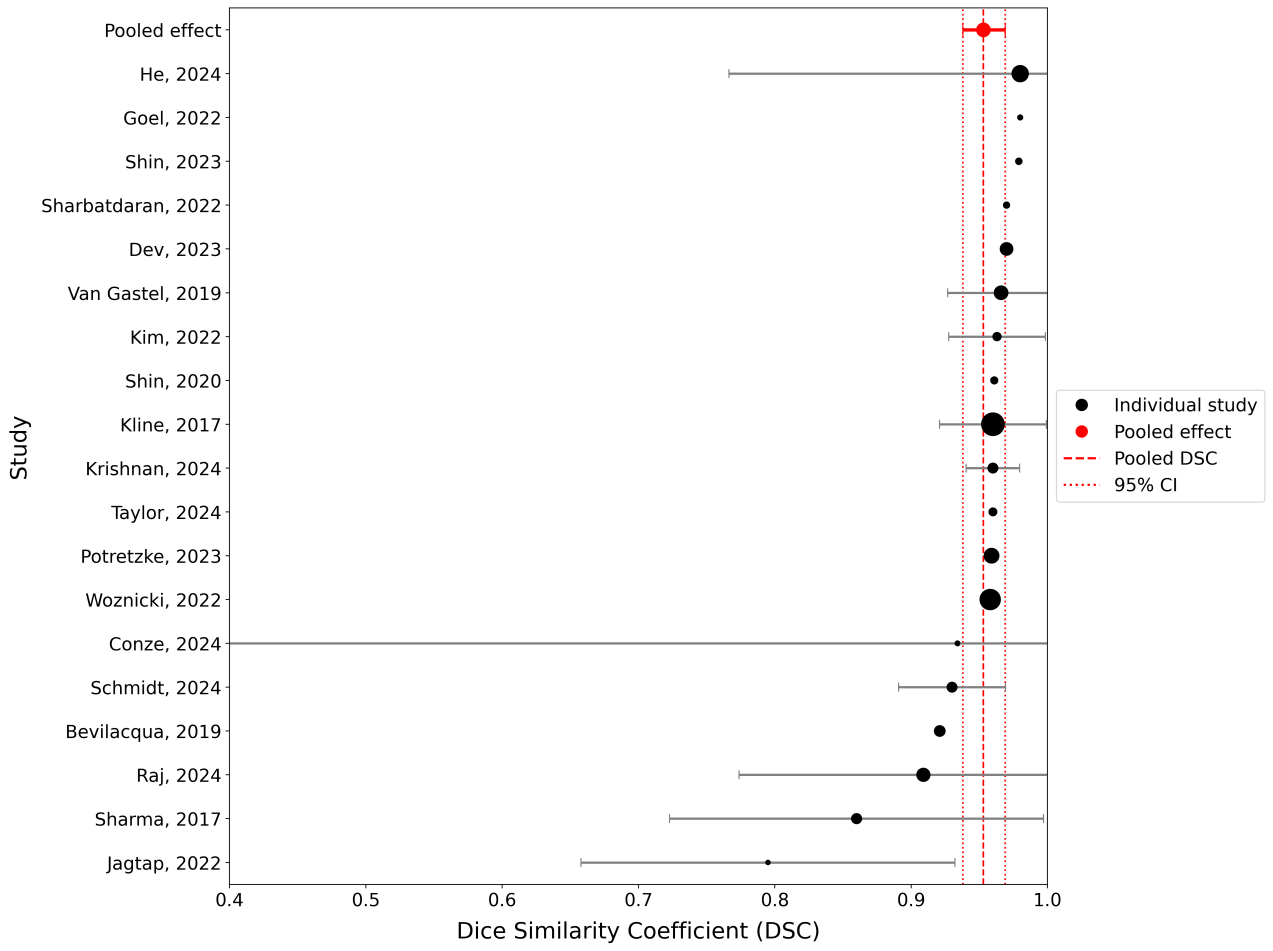
Forest plot of Dice Similarity Coefficients (DSCs) across ADPKD segmentation studies [17,25,26,27,28,29,30,31,32,33,34,35,36,37,38,39,40,41,42]. Each bubble represents a study, with the bubble size proportional to the test sample size of the study. Horizontal gray error bars denote 95% confidence intervals (CI) for studies with available standard deviations. The red dashed vertical line indicates the pooled DSC calculated using a random-effects meta-analysis, and the red dotted lines represent the 95% CI of the pooled estimate.

A random-effects meta-analysis was conducted to synthesize the segmentation performance reported across eligible deep learning studies on ADPKD imaging. Ten studies were included after excluding those with insufficient variance data. The pooled DSC under the random-effects model was 0.953 (95% CI: 0.938–0.969), indicating consistently high segmentation accuracy across studies (Figure 2). The narrow confidence interval suggests limited heterogeneity and stable model performance despite differences in imaging modalities and architectures. Overall, the findings support the reliability of DL segmentation models for volumetric assessment of ADPKD.

Regarding imaging protocols, there was considerable heterogeneity among the studies that used MRI as an imaging modality, both in the type of sequences (e.g., T1, T2) and the imaging plane (e.g., axial vs. coronal). However, none of the studies used proton density sequences or the sagittal plane for image segmentation. Almost all studies were multi-center, as previously mentioned, but they were also multi-vendor, adding another layer of heterogeneity to the results. Sixteen percent of studies used a small percentage of non-ADPKD patients to develop their DL model. Regarding training and datasets, the test sets were, on average, 17% (4–32%) larger than the training set but ranged from 15 to 400 scans. Only 21% of studies used external datasets for testing, and only one study did not report the size of the testing set [25].

**Table 1:** Characteristics of included studies. CoI: conflicts of interest; DL: deep learning; GT: ground truth; MS: manual segmentation; N/A: not applicable; ND: nothing to disclose; NR: not reported; N/S: not specified; P: prospective; R: retrospective; RT: radiologic technologist; SAS: semi-automated segmentation; SD: standard deviation. ^1^ Studies with multiple datasets report the mean of means. ^2^ Includes renal cyst patients without ADPKD. ^3^ Estimate by authors.

Study and Patient Characteristics								DL Model Characteristics			Other
First Author, Year	Type of Study	Dataset Source	Modality	No. of Scans	No. of ADPKD Patients	Age (Mean ± SD/Range) ^1^	Sex (% Male) ^1^	Reference Standard	Segmentation Model	Time Efficiency (GT vs. Model; Default Unit: min)	Conflicts of Interest
Kline, 2017 [33]	R	TEMPO Study, Multi-center, Global	MRI	2400	N/S	N/S	N/S	Clinical expert	SAS Multi-observer CNN	N/A	NR
Sharma, 2017 [35]	R	Multi-study, Multi-center, USA	CT	244	125	51.9 [28–74]	53%	Clinical expert	MS 2D-CNN	∼30 vs. <1 ^3^	ND
Bevilacqua, 2019 [37]	R	Single-center, IT	MRI	18	18	31.3 ± 15.5	N/S	Radiologist	MS Semantic CNN	N/A	ND
van Gastel, 2019 [38]	R	DIPAK-1 Study, Multi-center, NL	MRI	585	540	49.1 ± 7.4	45%	MS	Semantic CNN	N/A	ND
Shin, 2020 [34]	R	Multi-center, KR	CT	214	214	N/S	N/S	Clinical expert	MS 3D V-Net	3.77 vs. 0.0072 min. per slice	ND
Goel, 2022 [26]	R, P	Multi-center, USA	MRI	286	173	47.1 ± 13.8	48.5%	Radiologist	MS 2D U-Net + EfficientNet	28.7 vs. 12.1	2 authors CoI
Jagtap, 2022 [39]	R	Single-center, USA	US	132	22	51 [28–70]	36.4%	Radiologist	SAS 2D U-Net	N/A	ND
Kim, 2022 [40]	R	HALT-PKD Trials, Multi-center, USA	MRI	210	210	37.9 ± 8.7	47.1%	Radiologist	MS 3D U-Net	N/A	1 author CoI
Sharbatdaran, 2022 [27]	R, P	Multi-center, USA	MRI	320	275	48.7 ± 14.3	45.8%	Radiologist	MS 2D U-Net + EfficientNet	Right: 7.65 vs. 4.52; Left: 7.57 vs. 4.27	2 authors CoI
Woznicki, 2022 [28]	R, P	Multi-study (DIPAK-1), Multi-center, Europe	MRI	2173	743	45.7 ± 10.7	44.3%	Radiologist	MS 2D and 3D U-Net	N/A	2 authors CoI
Shin, 2023 [30]	R, P	Multi-center, KR	CT	753	753	N/S	N/S	Radiologist	MS 3D U-Net, weighted loss	N/A	ND
Dev, 2023 [29]	R, P	Multi-center, USA	MRI	471	413 (454 ^2^)	48.7 ± 14.1	46%	Radiologist	MS 2D U-Net + EfficientNet	11.57 vs. 2.82	NR
Potretzke, 2023 [25]	P	Multi-center, USA	MRI	170	161	45.2 ± 14.5	34.7%	Radiologist + RT	SAS 2D-CNN	N/A	2 authors CoI
Conze, 2024 [41]	R	Genkyst Cohort, Multi-center, FR	MRI	118	112	47.1 ± 14.2	41.5%	Nephrologist	MS SwinU-NetV2	N/A	ND
He, 2024 [31]	R, P	Multi-center, USA	MRI	1429	470 (494 ^2^)	46 (IQR 37–55)	46%	Radiologist	MS Multimodal 3D U-Net	Right: 9.22 vs. 0.78; Left: 9.73 vs. 0.77	2 authors CoI
Krishnan, 2024 [32]	R, P	CRISP Study, Multi-center, USA	MRI	756	95	N/S	N/S	Clinical expert	SAS 3D U-Net	N/A	2 authors CoI
Raj, 2024 [17]	R	CRISP Study, Multi-center, USA	MRI	270	135	32 ± 9	43%	MS + radiologist	Attention 2D U-Net	N/A	ND
Schmidt, 2024 [42]	R	CRISP Study, Multi-center, USA	MRI	756	95	N/S	N/S	MS	2D U-Net	N/A	2 authors CoI
Taylor, 2024 [36]	R	CYSTic Consortium, Multi-center, Europe	MRI	275	260	45.1 ± 12.2	46.2%	MS	Ensemble U-Net	56 vs. 8.5	ND

**Figure 3:**
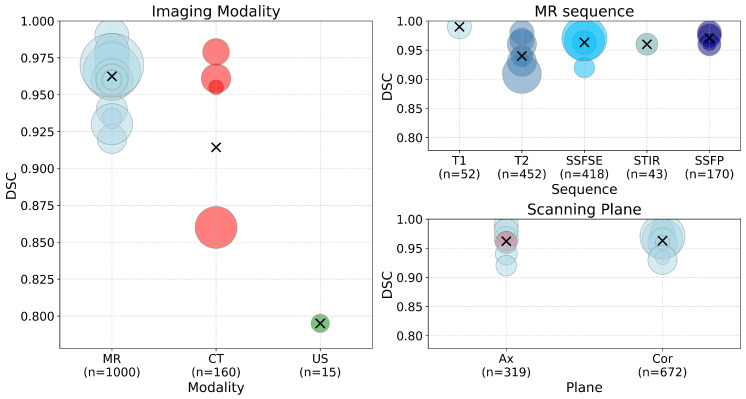
DSC performance by input imaging modality for deep learning-based kidney segmentation in ADPKD. Across the plots, blue represents MRI, red represents CT, and green represents US. The main panel displays the DSC values organized by imaging modality (MRI, CT, US), with the bubble size indicating the number of studies using each modality. The black ‘X’ markers denote the mean DSC value for each modality. The subplots on the right provide additional breakdowns: the top-right shows the DSC values by MRI sequence (T1, T2, SSFSE, STIR, SSFP), and the bottom-right shows the DSC values by scanning plane (axial, coronal).

Figure 3 summarizes the DSC values achieved by the selected methods for kidney segmentation across different factors. Subgroup analysis highlights the high performance of MRI, with decreased performance in CT and ultrasound modalities. Minor differences were observed between the various MRI sequences, with T2 exhibiting a lower average and a wider interval compared to single-shot fast spin echo (SSFSE), short tau inversion recovery (STIR), and steady-state free precession (SSFP). Scanning planes used for analysis showed no significant differences between them.

In this plot, the weighted mean μ of the DSC values was calculated using the number of scans $w_{i}$ as weights for each observation $x_{i}$, as given by

$$\mu =\frac{\sum_{i=1}^{n}w_{i}x_{i}}{\sum_{i=1}^{n}w_{i}}.$$

To quantify the variability of the weighted DSC values, the weighted variance $\sigma^{2}$ was computed as

$$\sigma^{2}=\frac{\sum_{i=1}^{n}w_{i}{\left(x_{i}-\mu \right)}^{2}}{\sum_{i=1}^{n}w_{i}},$$

from which the weighted standard deviation (σ) was obtained by a square root transformation. Error bars showing ±1 SD around the weighted mean were then plotted as

Lowerlimit=μ−σ,Upperlimit=μ+σ,

**Table 2:** Summary of included studies using various imaging modalities and deep learning models. Reported details include dataset size (with test set breakdown), percentage of ADPKD cases, model architecture, DSC with standard deviation, and mean group- or subject-level TKV bias. When RK and LK metrics are reported separately, it is assumed that the number of RK and LK samples is equal, and both values are averaged. The table is sorted in descending order of DSC values. When DSC values are reported for multiple cohorts, a weighted average is used. If two studies report identical DSC values, they are further sorted in descending order based on bias in TKV. ^1^ R refers to “Reproducibility Test Set”, E to “External Test Set”, and I to “Internal Test Set”. ^2^ When percentages of ADPKD cases are reported for both training and test sets, the test set percentage is shown in parentheses. If not specified, the paper reports only the overall percentage per dataset without providing a detailed breakdown of the split. ^3^ The calculation is a group-level bias (% difference of means). Bland–Altman individual-level bias reflects case-by-case differences, and its % bias often uses relative error per subject. ^4^ Split is only mentioned patient-wise and not scan-wise.

First Author, Year	Image Protocol	Dataset Size (Test Size) ^1^	Acquisition	%ADPKD ^2^	Model	DSC (±std)	Bias TKV Difference (%) ^3^
He, 2024 [31]	Ax (T1, T2, SSFP, DWI), Cor (T2, SSFP)	1429 (I:118/E:90)	Multi-vendor, multi-center	94% (100%)	Multimodal 3D U-Net	I:0.98 ± 0.04/ E:0.98 ± 0.2	0.57 (Absolute)
Goel, 2022 [26]	AxT2W SSFSE	286 (20)	Multi-vendor, multi-center	100%	2D U-Net, encoder EfficientNet	0.98	**(+)** 2.55
Shin, 2023 [30]	CT	753 (32)	Multi-vendor, multi-center	100%	3D U-Net, loss variably weighted	0.979	≈ **(+)** 0.78
Sharbatdaran, 2022 [27]	Ax (T2, DWI), Cor (T2, SSFP), SPGR, DixonFS	320 (E:30)	Multi-vendor, multi-center	100%	2D U-Net, encoder EfficientNet	0.97	**(+)** 5
Van Gastel, 2019 [38]	CorT2 SSFSE	585 (145)	Multi-center	100%	Semantic CNN	0.966 ± 0.02	(+) < **0.1**
Kim, 2022 [40]	CorT2W SSFSE	210 (53)	Multi-center	100%	3D U-Net	0.963 ± 0.0181	**(−)** 2.42 mL
Shin, 2020 [34]	AxCT	214 (39)	Multi-vendor, multi-center	100%	3D V-Net	0.961	**(−)** 0.158
Krishnan, 2024 [32]	CorT2W	756 (76)	Multi-vendor, single-center	100%	3D U-Net	0.96 ± 0.01	**(+)** 0.42
Kline, 2017 [33]	T2 SSFSE, T1 SPGR, TrueFISP	2000 (400)	Multi-vendor, multi-center	100%	Multi-observer CNN	0.96 ± 0.02	**(−)** 0.65
Taylor, 2024 [36]	SSFP	227 (48)	Multi-vendor, multi-center	100%	Ensemble U-Net	0.96	**(−)** 1.65
Potretzke, 2023 [25]	CorSSFSE	170	Single-center, multi-vendor	≥93.9%	2D-CNN	0.959	**(−)** 3.318
Woznicki, 2022 [28]	Ax (T2, SSFSE, SPIR), Cor (SSFSE, TRUFI)	2173 (I:324/E:831)	Multi-vendor, multi-center	100%	Ensemble of 2D and 3D U-Net	I: 0.958	**I: (−)** 1.52, **E:(−) 1.3**
Dev, 2023 [29]	Ax and Cor (T1W, T2W, SSFP)	802 (R:85/E:40)	Multi-vendor, multi-center	89.7% (100%)	2D U-Net, encoder EfficientNet	R:0.98 /E: 0.955	R: **(+) 0.37**
Conze, 2024 [41]	CorT2	118 (18)	Multi-vendor, multi-center	100%	2D SwinU-NetV2	0.934 ± 0.276	0.09 (Absolute)
Schmidt, 2024 [42]	CorT2W	756 (76)	Multi-vendor, multi-center	100%	2D U-Net	0.93 ± 0.02	-
Bevilacqua, 2019 [37]	Cor(STIR, T2W), T1W	526 (≈17%) ^4^	Single-vendor, single-center	100%	Semantic CNN	0.921	-
Raj, 2024 [17]	T2W	135 (20% 5-folds)	Multi-vendor, multi-center	100%	Attention 2D U-Net	0.909 ± 0.069	**(+)** 66.82 mL/m (HtTKV)
Sharma, 2017 [35]	CT	244 (79)	Multi-center	100%	2D-CNN	0.86 ± 0.07	**(+) 3.40**
Jagtap, 2022 [39]	3D US (B-Mode, 1–5 MHz)	66 (15)	Single-vendor, single-center	100%	2D U-Net	0.795 ± 0.07	**(−) 4.12**

**Figure 4:**
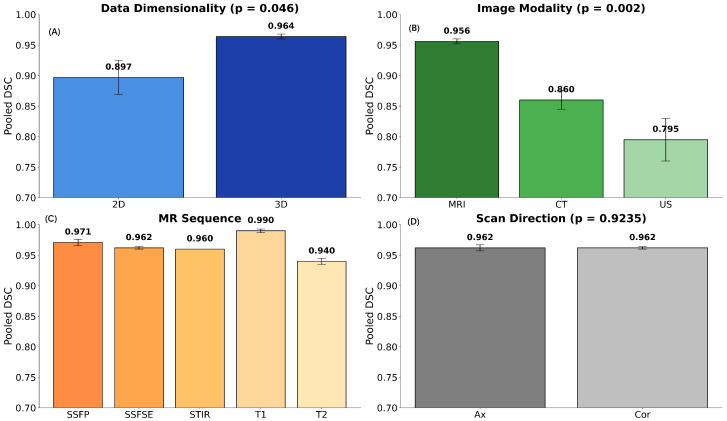
Meta-regression comparison of imaging parameters on segmentation performance. Pooled Dice Similarity Coefficient (DSC) values and 95% confidence intervals are shown for subgroups across (**A**) data dimensionality, (**B**) imaging modality, (**C**) MRI sequence, and (**D**) scan direction. Overall, these results indicate that 3D and MRI-based datasets, particularly T1, achieve superior performance.

A random-effects meta-regression (DerSimonian–Laird estimator) was conducted to account for between-study heterogeneity, with architecture dimensionality (2D vs. 3D) entered as a binary moderator. Across studies reporting the mean DSC, the pooled 2D mean DSC was 0.897 (95% CI: 0.869–0.926), while the pooled 3D mean DSC was 0.964 (95% CI: 0.960–0.969). The meta-regression showed that 3D architectures achieved significantly higher DSC values than 2D architectures on average (meandifference=0.062, 95% CI ≈[0.001,0.122], p=0.046two-sided; p=0.023one-sided). The standardized mean difference (Cohen’s d) was 1.00, indicating a large effect size. A post hoc power analysis (α=0.05 one-sided) revealed a statistical power of approximately 1.000, confirming that the analysis was sufficiently powered to detect the observed effect. Overall, these findings provide strong evidence that 3D deep learning architectures outperform 2D models in segmentation accuracy when study results are aggregated under a random-effects meta-analytic framework. The results are presented in Figure 4 (*top left*), alongside the other random-effects meta-regression analyses. They revealed a significant effect of imaging modality (*top right*) on segmentation performance (p=0.002). MRI-based models achieved markedly higher DSC values (mean=0.956) than CT (0.860) and US (0.795), with a large effect size for MRI vs. CT (Cohen’sd=1.42, power=1.00). Pooled DSC values across MRI sequence types (*bottom left*) showed that T1-weighted images achieved the highest segmentation performance (0.990, 95% CI: 0.987–0.993), followed by SSFP (0.971, 95% CI: 0.966–0.975), SSFSE (0.962, 95% CI: 0.960–0.964), and T2-weighted images (0.940, 95% CI: 0.935–0.944). STIR sequences had a pooled DSC of 0.960, but no confidence interval could be calculated because only one study reported a mean DSC, and no standard deviation was available for this group. A random-effects meta-regression using T1 as the reference category indicated that T2 sequences were significantly lower than T1 (meandifference=−0.028, p=0.040). In contrast, differences for SSFSE (meandifference=−0.018, p=0.192), SSFP (meandifference=−0.021, p=0.370), and STIR (*p* not estimable) were not statistically significant. These results suggest that T1-weighted sequences tend to provide higher segmentation accuracy, while other sequences show comparable performance; however, STIR could not be formally tested due to limited data. Pooled DSC values for imaging planes (*bottom right*) were very similar, with axial images achieving 0.962 (95% CI: 0.957–0.968) and coronal images achieving 0.962 (95% CI: 0.960–0.964). A random-effects meta-regression using axial as the reference showed no significant difference between coronal and axial planes (meandifference=0.001, p=0.924), suggesting that segmentation accuracy was essentially equivalent for the two orientations.

### 3.4. Sensitivity and Heterogeneity Analysis

Sensitivity analyses demonstrated the robustness of the pooled estimate. Excluding the lowest-performing 10% of studies did not materially alter the results, with the pooled DSC remaining at 0.953, which is identical to the estimate from the entire model. This consistency indicates that no single study disproportionately influenced the overall outcome. The Q test yielded a value of 2.02, and the Higgins *I*^2^ statistic showed a 0.00% variance. This suggests that the methods performed similarly across the included test sets. Although the between-study heterogeneity statistic was low (*I*^2^ = 0%), this may reflect ceiling effects inherent to DSC metrics, where values approach the upper limit of 1. To provide a more informative assessment, a 95% prediction interval was calculated, ranging from 0.931 to 0.975, suggesting that future studies should report DSC values within this high-performance range. Overall, these findings confirm the stability, accuracy, and reproducibility of DL segmentation performance across ADPKD imaging studies.

### 3.5. Methodological Quality Assessment

**Figure 5:**
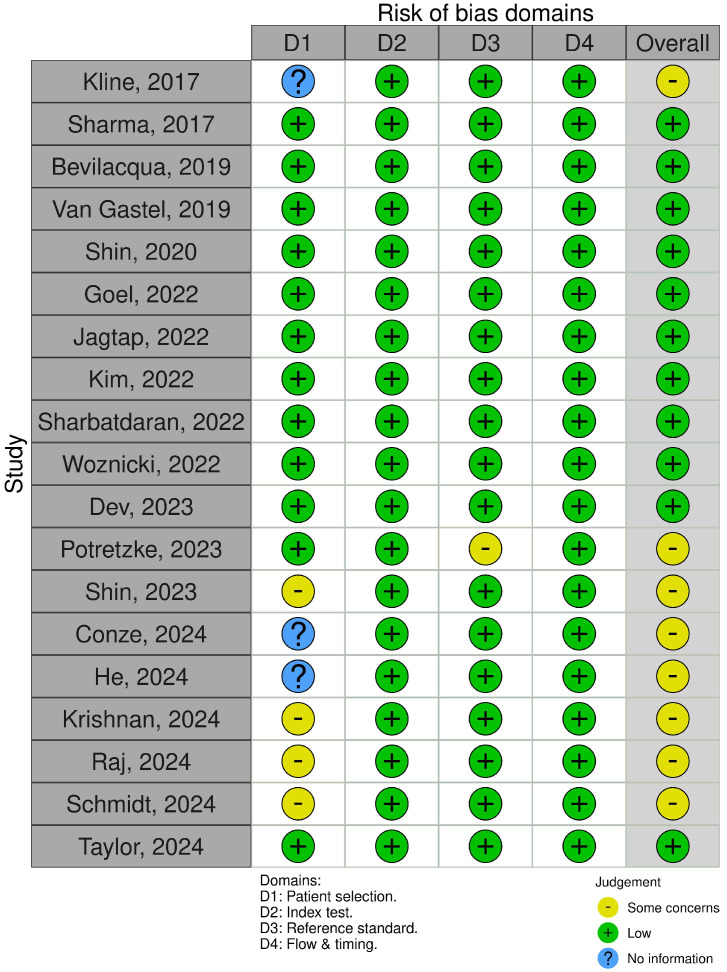
Schematic presentation of the risk of bias for the articles evaluated using QUADAS-2 [17,25,26,27,28,29,30,31,32,33,34,35,36,37,38,39,40,41,42]. Table made using the RobVis tool provided by Ref. [43].

The included studies demonstrated an overall low risk of bias, although some concerns were noted in certain articles. Figure 5 provides an overview of the quality assessment of the included studies using the QUADAS-2 tool. Concerning bias risk in patient selection, three studies [31,33,41] exhibited an unclear risk due to a missing description of inclusion and/or exclusion criteria. Four studies raised some concerns [17,30,32,42] due to exclusion criteria (e.g., exclusion of patients with TKV above 600 mL), resulting in a homogeneous patient group with only milder degrees of disease. However, there were no concerns regarding the index test, flow, and timing in the included studies. One study raised concerns about the ground truth [25] because manual segmentation was performed either by accepting the index test if deemed sufficient or by editing the index test if there were areas of discrepancy. However, this may simulate real-world use of such a tool.

The degree of fulfillment of information for the different items in CLAIM is shown in Table 3. The studies presented an average CLAIM score of 26.7 (64%), with an SD of 4.6, and scores between 17 and 35 out of a possible 42. The most frequently underreported subsections were data (55%), ground truth (58%), and training (46%) from methods, with almost half of the included articles failing to provide sufficient information. For the remaining subsections, at least two-thirds of the articles provided the required information.

The mean scores and SD (%) of CLAIM’s subsections were title/abstract at 1.58 ± 0.61 (80%), and introduction consistently at 1.95 ± 0.23 (97%). This means that the majority of authors identified the study as an AI methodology, specified the technology used (e.g., deep learning), and provided enough scientific/clinical background in the introduction, including the intended use and role of the AI approach. Overall, the methods section was underreported according to the checklist, with an average of 16.26 ± 3.69 out of 28 (59%). This section was broken down into study design (1.37 ± 0.50, 68%), data (3.84 ± 0.96, 55%), ground truth (2.89 ± 0.99, 58%), data partition (1.95 ± 0.40, 65%), model (1.89 ± 0.99, 63%), training (1.37 ± 0.90, 46%), and evaluation (2.95 ± 1.39, 59%). This means that the study goal, inclusion and exclusion criteria, selection of data subsets, de-identification of data, handling of missing data, rationale for choosing the reference standard, measures of inter- and intra-rater variability of features described by the annotators, intended sample size, initialization of model parameters, ensembling techniques, robustness or sensitivity analysis, methods for explainability or interpretability, and testing on external data were commonly not reported. For the results section, the mean was 3.42 ± 0.84 (71%) out of a total of five possible points. It was subdivided into data (1.63 ± 0.50, 82%) and model performance (1.79 ± 0.54, 60%), indicating that performance metrics, measures of statistical uncertainty, and failure analysis of incorrect results were partially or not reported in up to one-third of the included articles. The discussion section scores were 1.74 ± 0.56 (87%). The other information section scores were 1.74 (58%), which means that the study limitations and the implications for practice, including the intended use and/or clinical role of the developed models, were often reported, but not the access or reference for the whole study protocol, additional technical details, and/or the statement for the availability of the software, trained model, and/or data.

### 3.6. Assessment of Publication Bias

To assess the presence of publication bias in studies reporting on the segmentation performance of deep learning (DL) models for autosomal dominant polycystic kidney disease (ADPKD), we performed Egger’s regression test using a logit-transformed Dice Similarity Coefficient (DSC). Given that DSC values are bounded between 0 and 1, a logit transformation was applied to linearize the metric and reduce skewness due to ceiling effects often observed in high-performing models. Specifically, DSC values were clipped to the range [0.001, 0.999] and transformed as

$$\mathrm{logit}(\mathrm{DSC})=\log\left(\frac{\mathrm{DSC}}{1-\mathrm{DSC}}\right).$$

**Figure 6:**
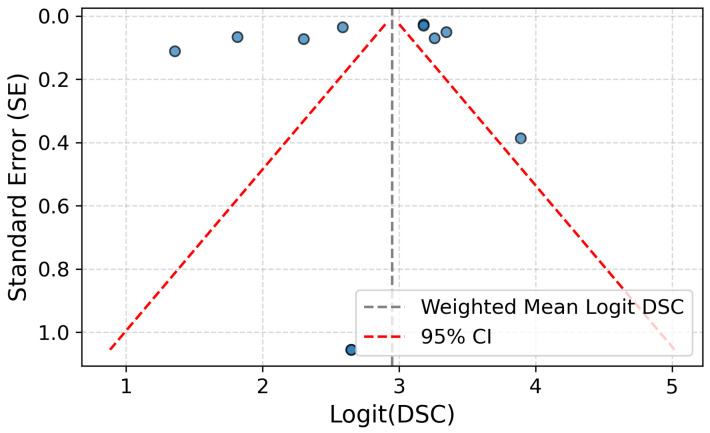
Funnel plot of DSC values for deep learning-based kidney segmentation in ADPKD.

The resulting funnel plot (Figure 6) of logit-transformed DSC values shows a symmetrical distribution around the weighted mean logit DSC of 2.94 (approximately 0.95 when back-transformed). Egger’s test on the transformed data yielded an intercept of −0.287 with a *p*-value of 0.783, indicating no statistically significant evidence of small-study effects or publication bias.

Although most studies appear outside the 95% confidence triangle in the funnel plot, this pattern is not necessarily indicative of publication bias. Rather, it likely reflects true heterogeneity across studies—such as differences in imaging protocols, model architectures, and dataset composition—as well as a narrow range of standard errors, which can make the funnel’s confidence region overly tight. The logit transformation mitigates ceiling effects but does not fully eliminate the influence of underlying variability. Given the non-significant results of Egger’s test, the observed dispersion is more plausibly attributed to methodological differences than to selective reporting.

## 4. Discussion

In patients with autosomal dominant polycystic kidney disease, total kidney volume is the gold standard biomarker for assessing the risk of progression and the need for drug therapy. However, manual kidney segmentation is a time-consuming process. The present systematic review and meta-analysis addressed two key questions: (1) whether image-based DL models for kidney volume assessment in polycystic kidney disease are ready for clinical deployment, and (2) what factors affect their generalization ability. In this study, we have shown that clinical implementation of these image-based DL segmentation models is not yet feasible, but the evidence supports their technical and diagnostic efficacy. First, we found that the pooled DSC was 0.953 (95% CI: 0.938–0.969) with relatively low bias for TKV in the 19 included studies (5622 ADPKD patients and 9180 scans). Second, the included models achieved an average reduction in segmentation time of 75% compared to standard segmentation. Third, in the subgroup analysis, we found that the MRI modality outperformed CT and US, with a slight difference in the type of MRI sequence, and no difference in the scanning plane used. Furthermore, 3D DL segmentation models outperformed 2D models. Lastly, no statistically significant evidence of inter-study heterogeneity, small-study effects, or publication bias was detected.

These findings are of clinical importance, as TKV measurements are used to determine disease progression, assess patient eligibility for treatments such as tolvaptan, and predict the need for dialysis. Automated kidney segmentation and calculation of TKV are faster and more accurate with image-based DL models, as demonstrated here. Utilizing DL segmentation models for clinical use could also advance research for patients with ADPKD. They have the potential to facilitate the identification and validation of new image-derived biomarkers that can help predict disease progression and response to therapy (for example, cyst volume measurement), and to assess the effectiveness of new therapies by comparing TKV measurements before and after treatment. TKV is used as a primary endpoint in clinical trials, so minimizing bias in measurements is critical for ensuring that trial results are reliable and reproducible. The low TKV bias with image-based DL segmentation models shown in this study supports the improvement of the clinical trial reliability of TKV.

To the best of our knowledge, this is the first systematic review and meta-analysis of image-based DL segmentation models for ADPKD patients. A previous article [27] reviewed and summarized several of the articles included here, but it was not a systematic review, nor was any meta-analysis of the data performed. Importantly, the current systematic review evaluated these DL segmentation models on large, representative populations with heterogeneous study designs (retrospective/prospective, multi-center) and methods (multi-vendor, multi-modality, multi-sequence, large training sets, prospective, internal and external dataset testing) that allowed for subgroup analysis, which is necessary before clinical deployment. Based on the meta-regressions, we were able to determine that MRI is the modality of choice and that 3D segmentation models outperform 2D models. Regarding MRI sequences, the analysis revealed that T1-weighted images achieve the highest segmentation performance; however, this finding was based on a single study with a small sample size (n=52). Additionally, SSFSE, STIR, and SSFP were superior to other T2-weighted images used for segmentation. We also identified one factor that did not influence the analyzed DL segmentation models: the selection of the scanning plane for MRI. However, further subgroup analysis was hindered by the underreporting of details and data in both the methodology and results sections of the included articles, as indicated by the CLAIM quality assessment. CLAIM was published in 2020, and among the 19 included studies, five were published in 2020 or earlier, which explains some of the underreporting. We encourage future work in medical applications of AI to consider using CLAIM to ensure reproducibility and transparency. To improve future studies, the editors and peer reviewers of journals should mandate checklist adherence through journal policies, implement a rigorous pre-publication review process to verify checklist completion, and promote open science practices, such as sharing code and data, to increase transparency and reproducibility [45]. Additionally, editors and peer-reviewed journals will need to adapt to this type of research, as it involves the use of massive amounts of data and numerous steps before AI models are developed and analyzed. Most journals have limits on the length of a manuscript, leading to missing data that could hinder the process and obstruct the potential translation of these studies into clinical settings, as well as their adequate evaluation [46].

### 4.1. Factors Influencing Performance

One compelling result was the significant time reduction achieved by the DL models compared to the standard measurement method. While the average reduction was substantial, the range of the reported times was wide. Some articles used fully automatic segmentation, while others employed semi-automatic segmentation, which could account for the differences in time efficiency. Although there could be other reasons for the variations, all would be speculative, as most articles did not provide detailed descriptions of these measurements. The reporting of these important metrics should be standardized to allow for comparison between DL models.

Of the tested MRI sequences, T1-weighted sequences outperformed T2-weighted sequences in the segmentation task, but on the one hand, there was a small amount of data available, and on the other hand, many studies only reported the DSC for one of the sequences in the imaging protocol, limiting the power of the pooled analysis. Very few tested the repeatability of the segmentation task based on the type of sequence used. SSFSE, STIR, and SSFP showed higher DSCs when compared to other T2-weighted sequences. These sequences share the common goal of achieving fast image acquisition through k-space traversal strategies but differ in their fundamental principles. They are all used to achieve specific high tissue contrasts and reduce motion artifacts compared to conventional spin-echo sequences, which have a clear advantage over the other T2 sequences. This is because the kidneys move due to breathing, which plays a role in the effectiveness of the DL model and explains the higher DSC in some of the T2-weighted image sequences.

An expected finding was the difference in DSC performance between 3D and 2D models in the meta-regression analysis. Three-dimensional models can better capture the spatial relationships between different parts of an object or structure within a volume, thereby reducing overfitting, but this comes at a higher computational cost. However, it must be noted that 2D models did not perform poorly and actually achieved good results (DSC: 0.897; 95% CI: 0.869–0.926). One reason for this finding is that renal segmentation is a task with well-defined boundaries, where 2D models are sometimes sufficient to perform the task. This may be explained by the anisotropic resolution of CT and MRI scans, where the in-plane resolution in the axial plane is typically ≈1, while the slice thickness in the axial direction is 3–5 mm. If the z-axis resolution is poor, the additional volumetric context captured by 3D models may not provide much beyond what 2D slices already capture. In addition, the large size and relatively smooth boundaries of polycystic kidneys allow accurate segmentation slice by slice without requiring extensive 3D spatial context. This may be explained by the large, smooth, and well-defined cystic morphology of polycystic kidneys, where most relevant information is already captured in-plane, making 2D approaches nearly as effective as 3D ones. Clinicians considering the implementation of these models could benefit from 2D models in clinical settings, as they are computationally less expensive and require less memory and processing power, making them suitable for resource-constrained environments and thus allowing for quicker development and deployment. They are also generally easier to implement and debug compared to 3D models.

### 4.2. Future Directions

For patients affected by ADPKD, successful differentiation of cysts is useful for automatic classification of patient phenotypes, clinical decision-making, and disease progression. The development of specific cyst segmentation and its volume measurement [16,47] can become critical biomarkers for tracking disease progression and assessing treatment efficacy in ADPKD patients, potentially surpassing the current gold standard of TKV. With the increasing complexity of DL segmentation models, the complexity of the task can also be increased, including cystic volume segmentation. These new models would allow tracking cyst growth. By tracking cyst growth, researchers can correlate cyst characteristics with specific genetic mutations (e.g., PKD1 or PKD2) to better understand the disease’s underlying mechanisms, thereby facilitating the study of genotype–phenotype correlations [16,48].

### 4.3. Strengths and Limitations

One major limitation of the current data is the lack of external validation, as only 21% of the studies provided this information. This limitation suggests that the models may not be reliable for broad clinical deployment, as their performance is likely specific to the data on which they were developed and could be overly optimistic. While clinical implementation is not yet feasible, the current work shows that image-based DL segmentation models are highly stable, accurate, and reproducible. In other words, the evidence supports their technical and diagnostic efficacy, and research in this area can move forward to prospective clinical trials to assess patient-centered outcomes, as well as observational and case studies to demonstrate real-world impact [49,50]. These studies must include performance metrics on new datasets, bias assessment, and analysis of long-term model longevity and performance. Clinical decision-makers cannot confidently use these tools without external validation, which is necessary to confirm real-world applicability and prevent potential compromises to patient safety [51]. And without proper validation, resources and effort spent on developing models may be wasted if they are not suitable for new contexts.

Other primary barriers to routine AI use include regulatory and workflow-related issues. Regulatory and ethical barriers include the following: (a) data privacy, as AI systems often require large amounts of sensitive personal data, raising significant concerns about privacy, security, and how that data is collected, used, and protected; (b) patient safety and liability, for instance, concerns about patient safety and who is liable if an AI makes an incorrect diagnosis or recommendation; and (c) regulations and standards, such as guidelines for development and deployment. The EU’s AI framework, primarily the AI Act, is the world’s first comprehensive legal framework for artificial intelligence, designed to balance innovation with safety and fundamental rights. It takes a risk-based approach, categorizing AI systems into four tiers: unacceptable, high, limited, and minimal risk. Unacceptable-risk applications, like social scoring and manipulative AI, are banned, while high-risk systems, which include all medical-related AI models, face strict requirements for providers and deployers [52]. Finally, the integration of AI algorithms (including image-based DL segmentation models) requires regulatory frameworks. Overall, regulatory bodies should ensure the compliant and safe use of DL models while facilitating innovation.

Workflow and organizational barriers include the following: (a) Workflow integration, as embedding AI tools into a team’s existing workflows requires significant redesign and adaptation. Once DL models are implemented into the radiology workflow, they may need to accommodate different radiologist and clinician preferences, various institutional practices, and the specific requirements of varying imaging machines or clinical contexts [53]. The implications of these are widely unknown. (b) A lack of AI expertise and training, which is required for the development, implementation, and management of AI systems in clinical settings. (c) The costs of implementing, maintaining, and monitoring AI systems. Additionally, organizations will struggle to demonstrate clear socio-economic benefits or business value [54].

AI is being rapidly integrated into society and medicine, and the use of this technology and its findings must be interpreted considering several limitations. First, the data suggest that ample and heterogeneous datasets (both patient-specific and scanning characteristics) were used for both training and validation in the included studies. However, the DL segmentation models themselves could start to diverge during deployment due to changes in image acquisition protocols, disease patterns, and continuous model updates. This phenomenon could lead to data and model drift, which involves a deterioration in model performance over time and necessitates constant monitoring of model outputs [55,56]. Second, due to their complexity and scale, the hardware requirements for training, deploying, and monitoring DL models pose financial and environmental challenges that hospitals, institutions, and private clinics must consider before clinical deployment. Third, the current PRISMA guidelines and CLAIM are not task-specific and lack standardization regarding the methods required to evaluate clinical DL models. For segmentation tasks, metrics such as Dice score and Hausdorff distance are often reported, but biases or confidence intervals are not. There is a clear need to develop a tailored PRISMA for AI [57] that also considers the importance of task-specific performance metrics. Fourth, as AI algorithms, including DL models, become increasingly trusted for real-world applications, there is a risk of overreliance on the models. This could result in automation bias and lead to incorrect segmentation, which in turn could lead to inaccurate diagnoses and/or treatment [58]. It is essential to establish best practices for the use and education of these tools before deployment to minimize this bias.

## 5. Conclusions

In summary, current deep learning algorithms for kidney volumetry in ADPKD patients have shown high precision compared to manual segmentation by experts in a large, pooled sample with heterogeneous study designs (retrospective/prospective, multi-center) and methods (multi-vendor, multi-modality, multi-sequence, and large training sets). Therefore, while clinical implementation is not yet feasible due to a lack of external validation, the current work shows that image-based DL segmentation models are highly stable, accurate, and reproducible, with a “high degree of certainty” based on the GRADE framework.

This systematic review and meta-analysis also showed that clinical applicability can be enhanced by using the MRI modality over CT and US. There were no differences regarding the scanning plane, and the results suggest that T1-weighted imaging outperformed T2-weighted imaging. If researchers or clinicians utilize T2-weighted imaging, they might benefit from using sequences that employ fast image acquisition through k-space traversal strategies (such as STIR or SSFP). However, this finding must be interpreted with caution due to the limited data available. Furthermore, the 3D DL segmentation models outperformed the 2D models.

## Figures and Tables

**Table 3:** Schematic presentation of the Checklist for Artificial Intelligence in Medical Imaging (CLAIM) [44].

	Title/ Abstract	Intro- duction	Methods							Results		Discussion	Other Information	Total Score by Author
			Study Design	Data	Ground Truth	Data Partition	Model	Training	Evaluation	Data	Model Performance			
First Author, Year	2 (n)	2 (n)	2 (n)	7 (n)	5 (n)	3 (n)	3 (n)	3 (n)	5 (n)	2 (n)	3 (n)	2 (n)	3 (n)	(42) N (%)
Kline, 2017 [33]	2	2	1	4	2	2	2	3	2	1	1	2	1	25 (61%)
Sharma, 2017 [35]	2	2	1	4	2	2	1	1	2	1	1	2	3	24 (57%)
Bevilacqua, 2019 [37]	1	2	1	3	1	2	1	1	1	1	1	1	1	17 (41%)
Van Gastel, 2019 [38]	0	2	1	3	2	1	0	0	4	2	2	2	2	21 (50%)
Shin, 2020 [34]	2	2	1	4	3	3	2	1	2	1	2	2	1	26 (62%)
Goel, 2022 [26]	2	2	1	5	3	2	2	2	5	2	2	2	3	33 (79%)
Jagtap, 2022 [39]	1	2	1	4	4	2	2	1	3	2	2	2	1	27 (64%)
Kim, 2022 [40]	2	2	1	5	3	2	1	1	2	2	2	2	1	26 (62%)
Sharbatdaran, 2022 [27]	2	1	1	3	3	2	2	1	5	2	2	2	2	28 (67%)
Woznicki, 2022 [28]	1	2	2	5	4	2	3	3	5	2	2	2	2	35 (84%)
Dev, 2023 [29]	1	2	1	2	4	2	3	1	4	2	2	2	1	27 (64%)
Potretzke, 2023 [25]	2	2	2	2	3	1	0	0	2	2	1	2	3	22 (52%)
Shin, 2023 [30]	2	2	2	5	3	2	3	2	2	1	2	2	3	31 (75%)
Conze, 2024 [41]	1	2	2	4	3	2	3	1	2	2	1	0	1	24 (57%)
He, 2024 [31]	2	2	2	3	2	2	2	1	5	2	2	2	3	30 (71%)
Krishnan, 2024 [32]	2	2	2	4	4	2	3	2	3	1	2	2	2	31 (74%)
Raj, 2024 [17]	1	2	1	4	4	2	1	1	1	2	2	1	1	23 (55%)
Schmidt, 2024 [42]	2	2	2	4	1	2	3	1	2	1	2	1	1	24 (57%)
Taylor, 2024 [36]	2	2	1	5	4	2	2	3	4	2	3	2	1	33 (79%)
**Total score by section (%)**	30 (80%)	37 (97%)	26 (68%)	73 (55%)	55 (58%)	37 (65%)	36 (63%)	26 (46%)	56 (59%)	31 (82%)	34 (60%)	33 (87%)	33 (58%)	-

## Data Availability

The original contributions presented in this study are included in the article. Any other data supporting this study’s findings are available from the corresponding author upon reasonable request.

## Abbreviations

The following abbreviations are used in this manuscript:

ADPKD	autosomal dominant polycystic kidney disease
AI	artificial intelligence
CLAIM	Checklist for Artificial Intelligence in Medical Imaging
CT	computer tomography
DL	deep learning
DSC	Dice Similarity Coefficient
eGFR	estimated glomerular filtration rate
ESRD	end-stage renal disease
FM	foundation models
GT	ground truth
MRI	magnetic resonance imaging
PRISMA	Preferred Reporting Items for Systematic Reviews and Meta-Analyses
QUADAS-2	Quality Assessment of Diagnostic Accuracy Studies, Version 2
SD	standard deviation
SSFP	steady-state free precession
SSFSE	single-shot fast spin echo
STIR	short tau inversion recovery
TKV	total kidney volume
US	ultrasound

## Appendix A. PRISMA 2020 Checklist

**Table A1:** PRISMA 2020 checklist: comprehensive set of recommended items to consider and report when conducting a systematic review, aimed at improving transparency, completeness, and reproducibility of the review process.

Topic	Item #	Checklist item	Location
**TITLE**			
Title	1	Identify the report as a systematic review.	Page 1
**ABSTRACT**			
Abstract	2	See the PRISMA 2020 for Abstracts checklist.	Page 1
**INTRODUCTION**			
Rationale	3	Describe the rationale for the review in the context of existing knowledge.	Page 1
Objectives	4	Provide an explicit statement of the objective(s) or question(s) the review addresses.	Page 2
**METHODS**			
Eligibility criteria	5	Specify the inclusion and exclusion criteria for the review and how studies were grouped for the syntheses.	Page 3
Information sources	6	Specify all databases, registers, websites, organizations, reference lists, and other sources searched or consulted to identify studies. Specify the date each source was last searched or consulted.	Page 2
Search strategy	7	Present the full search strategies for all databases, registers, and websites, including any filters and limits used.	Pages 2–3, Appendix B
Selection process	8	Specify the methods used to decide whether a study met the inclusion criteria of the review, including how many reviewers screened each record and each report retrieved, whether they worked independently, and, if applicable, details of the automation tools used in the process.	Page 3
Data collection process	9	Specify the methods used to collect data from reports, including how many reviewers collected data from each report, whether they worked independently, any processes for obtaining or confirming data from study investigators, and, if applicable, details of the automation tools used in the process.	Page 3
Data items	10a	List and define all outcomes for which data were sought. Specify whether all results that were compatible with each outcome domain in each study were sought, and if not, the methods used to decide which results to collect.	Pages 3–4
	10b	List and define all other variables for which data were sought (e.g., participant and intervention characteristics, funding sources). Describe any assumptions made about any missing or unclear information.	Pages 3–4
Study risk of bias assessment	11	Specify the methods used to assess risk of bias in the included studies, including details of the tool(s) used, how many reviewers assessed each study, whether they worked independently, and, if applicable, details of the automation tools used in the process.	Page 3
Effect measures	12	Specify for each outcome the effect measure(s) (e.g., risk ratio, mean difference) used in the synthesis or presentation of results.	NA
Synthesis methods	13a	Describe the processes used to decide which studies were eligible for each synthesis.	Page 4
	13b	Describe any methods used to prepare the data for presentation or synthesis.	Page 6
	13c	Describe any methods used to tabulate or visually display the results of individual studies and syntheses.	Pages 6, 7, 9, 10
	13d	Describe any methods used to synthesize results and provide a rationale for the choice(s). If meta-analysis was performed, describe the model(s), method(s) used to identify the presence and extent of statistical heterogeneity, and the software package(s) used.	Page 4
	13e	Describe any methods used to explore possible causes of heterogeneity among study results (e.g., subgroup analysis, meta-regression).	Pages 4, 6, 7, 10
	13f	Describe any sensitivity analyses conducted to assess the robustness of the synthesized results.	NA
Reporting bias assessment	14	Describe any methods used to assess risk of bias due to missing results in a synthesis (arising from reporting biases).	Pages 4, 13
Certainty assessment	15	Describe any methods used to assess certainty (or confidence) in the body of evidence for an outcome.	Pages 4, 6, 9, 13
**RESULTS**			
Study selection	16a	Describe the results of the search and selection process, from the number of records identified in the search to the number of studies included in the review, ideally using a flow diagram.	Pages 4–5
Study characteristics	17	Cite each included study and present its characteristics.	Table 1
Risk of bias in studies	18	Present assessments of risk of bias for each included study.	Figure 3, Table 3
	16b	Cite studies that might appear to meet the inclusion criteria but were excluded and explain why they were excluded.	Page 4
Results of individual studies	19	For all outcomes, report the following for each study: (a) summary statistics for each group (where appropriate), and (b) an effect estimate and its precision (e.g., confidence/credible interval). Ideally, present these data using structured tables or plots.	Page 6, Table 2, Figure 2
Results of syntheses	20a	For each synthesis, briefly summarize the characteristics and risk of bias among contributing studies.	Page 10–12
	20b	Present results of all statistical syntheses conducted. If a meta-analysis was conducted, for each, present the summary estimate and its precision (e.g., confidence/credible interval) and measures of statistical heterogeneity. If comparing groups, describe the direction of the effect.	Pages 6–8
	20c	Present results of all investigations of possible causes of heterogeneity among study results.	Pages 8–10 and Figure 3 and Figure 4
	20d	Present results of all sensitivity analyses conducted to assess the robustness of the synthesized results.	NA
Reporting biases	21	Present assessments of risk of bias due to missing results (arising from reporting biases) for each synthesis assessed.	Page 13, Figure 5
Certainty of evidence	22	Present assessments of certainty (or confidence) in the body of evidence for each outcome assessed.	Page 6
**DISCUSSION**			
Discussion	23a	Provide a general interpretation of the results in the context of other evidence.	Pages 14–16
	23b	Discuss any limitations of the evidence included in the review.	Pages 16–17
	23c	Discuss any limitations of the review processes used.	Pages 16–17
	23d	Discuss implications of the results for practice, policy, and future research.	Pages 15, 17
**OTHER INFORMATION**			
Registration and protocol	24a	Provide registration information for the review, including register name and registration number, or state that the review was not registered.	Page 2
	24b	Indicate where the review protocol can be accessed, or state that a protocol was not prepared.	Page 2
	24c	Describe and explain any amendments to information provided at registration or in the protocol.	NA
Support	25	Describe sources of financial or non-financial support for the review, and the role of the funders or sponsors in the review.	Page 18
Competing interests	26	Declare any competing interests of review authors.	Page 18
Availability of data, code, and other materials	27	Report which of the following are publicly available and where they can be found: template data collection forms; data extracted from included studies; data used for all analyses; analytic code; any other materials used in the review.	Page 18

## Appendix B. Complete Search Strategies and Syntax

### Appendix B.1. Eligibility Criteria

The PICO framework was used to structure the reporting of the eligibility criteria (Table A2).

**Table A2:** PICO elements and identification of search terms.

	PICO (T)	Our Elements ⇒ Search Terms
**P**	**POPULATION / PATIENT / PROBLEM** What is the patient population or primary problem? What are the relevant demographic factors or most important characteristics of the patient? What is the setting?	Patients with autosomal dominant polycystic kidney disease in humans of all ages and sexes.
**I**	**INTERVENTION (DIAGNOSIS)** What is the main intervention, treatment, diagnostic test, procedure, exposure, patient perception, or risk factor? What are the dosage, frequency, duration, and mode of delivery?	Imaging techniques and protocols that included computed tomography, magnetic resonance imaging, or ultrasound using deep learning applications and segmentation architectures.
**C**	**COMPARISON / CONTROL** *(optional)* Is there an alternative intervention or treatment to compare? Active: a different drug, dose, or kind of therapy Inactive: placebo, standard care, no treatment	Not applicable (radiologists as gold standard)
**O**	**OUTCOME** *(optional)* What is(are) the ideal clinical outcome(s)? It should be specific and measurable. It can be objective or subjective.	Kidney volume (height-adjusted) as a predictor of future kidney failure and an indicator for initiating treatment.
**T**	**TIME** *(optional)* How much time does it take to demonstrate the clinical outcome(s)?	Not applicable
**My literature search question is:**		Purpose: To examine kidney volume measurements by using image-based deep learning in patients with (an autosomal dominant) polycystic kidney disease compared to radiologists.

### Appendix B.2. Search Strategy

To develop a search strategy, a list of keywords and their synonyms was created based on the eligibility criteria and in the context of the databases used for the search (Table A3). An information specialist from Copenhagen University Library, Health/Science, was consulted during the process.

**Table A3:** List of keywords and their synonyms used to develop the search strategy.

Keywords	Synonyms
Imaging	**PubMed search:** (medical imaging) OR radiodiagnosis OR (CT scan) OR MRI OR ultrasound.
	**Elaboration of PubMed search including MeSH terms and all fields:**
	medical imaging: “radiography” [MeSH Terms] OR “radiography” [All Fields] OR (“medical” [All Fields] AND “imaging” [All Fields]) OR “medical imaging” [All Fields] OR “diagnostic imaging” [MeSH Terms] OR (”diagnostic” [All Fields] AND “imaging” [All Fields]) OR “diagnostic imaging” [All Fields]
	radiodiagnosis: “radiodiagnosis” [All Fields]
	CT scan: “tomography, x-ray computed” [MeSH Terms] OR (“tomography” [All Fields] AND “x-ray” [All Fields] AND “computed” [All Fields]) OR “x-ray computed tomography” [All Fields] OR (”ct” [All Fields] AND “scan” [All Fields]) OR “ct scan” [All Fields]
	MRI: “magnetic resonance imaging” [MeSH Terms] OR (“magnetic” [All Fields] AND “resonance” [All Fields] AND “imaging” [All Fields]) OR “magnetic resonance imaging” [All Fields] OR “mri” [All Fields]
	ultrasound: “diagnostic imaging” [Subheading] OR (“diagnostic” [All Fields] AND “imaging” [All Fields]) OR “diagnostic imaging” [All Fields] OR “ultrasound” [All Fields] OR “ultrasonography” [MeSH Terms] OR “ultrasonography” [All Fields] OR “ultrasonics” [MeSH Terms] OR “ultrasonics” [All Fields] OR “ultrasounds” [All Fields] OR “ultrasound’s” [All Fields]
	**Embase and Ovid MEDLINE search, including subject heading or keyword [.mp.]:** diagnostic imaging/ or “imaging and display”/ or radiodiagnosis/ or x-ray computed tomography/ or computer assisted tomography/ or nuclear magnetic resonance imaging/ or (diagnostic imaging or medical imaging or radiodiagnosis or (CT scan or ct scanning or x-ray computed tomography or computer assisted tomography) or (MRI or magnetic resonance imaging or mr imaging or nuclear magnetic resonance imaging or nmr imaging) or (ultrasonography or ultrasonography or echography or ultrasonogram or ultrasonic scanning or ultrasound scanning or ultrasound scan)).mp.
	**List of keywords:** diagnostic imaging, imaging and display, radiodiagnosis, medical imaging, CT scan, ct scanning, x-ray computed tomography, computer assisted tomography, MRI, magnetic resonance imaging, mr imaging, nuclear magnetic resonance imaging, nmr imaging, ultrasonography, echography, ultrasonogram, ultrasonic scanning, ultrasound scanning, ultrasound scan
Deep Learning	**PubMed search:** (Hierarchical Learning) OR (convolutional neural network)
	**Elaboration of PubMed search, including MeSH terms and all fields:**
	hierarchical learning: “deep learning” [MeSH Terms] OR (“deep” [All Fields] AND “learning” [All Fields]) OR “deep learning” [All Fields] OR (“hierarchical” [All Fields] AND “learning” [All Fields]) OR “hierarchical learning” [All Fields]
	convolutional neural network: (“convolute” [All Fields] OR ”convoluted” [All Fields] OR “convolutes” [All Fields] OR “convoluting” [All Fields] OR “convolution” [All Fields] OR ”convolutional“ [All Fields] OR ”convolutions” [All Fields] OR “convolutive” [All Fields]) AND (“neural networks, computer” [MeSH Terms] OR (“neural” [All Fields] AND “networks” [All Fields] AND “computer” [All Fields]) OR “computer neural networks” [All Fields] OR (“neural” [All Fields] AND “network” [All Fields]) OR “neural network” [All Fields])
	**Embase and Ovid MEDLINE search, including subject heading or keyword [.mp.]:** deep learning/ or deep neural network/ or convolutional neural network/ or convolution algorithm/ or (deep learning or hierarchical learning or deep neural network or convolutional neural network or convolution algorithm).mp.
	**List of keywords:** deep learning, hierarchical learning, convolutional neural network, convolution algorithm, deep neural network
Polycystic Kidney	**PubMed search:** ((polycystic kidney) OR (polycystic renal disease)) OR (ADPKD)
	**Elaboration of PubMed search, including MeSH terms and all fields:**
	polycystic kidney: “polycystic kidney diseases” [MeSH Terms] OR (“polycystic” [All Fields] AND “kidney” [All Fields] AND “diseases” [All Fields]) OR “polycystic kidney diseases” [All Fields] OR (“polycystic” [All Fields] AND “kidney” [All Fields]) OR “polycystic kidney” [All Fields]
	polycystic renal disease: ”polycystic kidney diseases“ [MeSH Terms] OR (”polycystic“ [All Fields] AND ”kidney“ [All Fields] AND ”diseases“ [All Fields]) OR ”polycystic kidney diseases“ [All Fields] OR (”polycystic“ [All Fields] AND ”renal“ [All Fields] AND ”disease“ [All Fields]) OR ”polycystic renal disease“ [All Fields]
	ADPKD: ”polycystic kidney, autosomal dominant“ [MeSH Terms] OR (“polycystic” [All Fields] AND “kidney” [All Fields] AND “autosomal” [All Fields] AND “dominant” [All Fields]) OR “autosomal dominant polycystic kidney” [All Fields] OR “adpkd” [All Fields]
	**Embase and Ovid MEDLINE search, including subject heading or keyword [.mp.]:** kidney polycystic disease/ or (polycystic kidney or renal polycystic disease or cystic kidney or cystic kidney disease or renal cystic disease or autosomal dominant polycystic kidney or ADPKD).mp.
	**List of keywords:** Kidney polycystic disease, polycystic kidney, renal polycystic disease, cystic kidney, cystic kidney disease, renal cystic disease, autosomal dominant polycystic kidney, ADPKD

The complete search history, with and without filters and limits for the electronic databases, is presented in Table A4 and Table A5:

**Table A4:** Search via PubMed (last search 13 October 2024).

Search Syntax	Results
((medical imaging) OR (CT scan) OR MRI OR ultrasound OR radiodiagnosis) AND ((Hierarchical Learning) OR (convolutional neural network)) AND ((polycystic kidney) OR (polycystic renal disease) OR ADPKD)	33
((medical imaging) OR (CT scan) OR MRI OR ultrasound OR radiodiagnosis) AND ((Hierarchical Learning) OR (convolutional neural network)) AND ((polycystic kidney) OR (polycystic renal disease) OR ADPKD) Filters: Danish, English, Humans, from 2000/1/1 - 2024/10/13	23

**Table A5:** Search via OvidSP platform, Embase <1974 to 11 October 2024>, and Ovid MEDLINE ALL <1946 to 13 October 2024>. (last search 13 October 2024).

Search Syntax	Results
1. diagnostic imaging/ or “imaging and display”/ or radiodiagnosis/ or x-ray computed tomography/ or computer assisted tomography/ or nuclear magnetic resonance imaging/ or (diagnostic imaging or medical imaging or radiodiagnosis or (CT scan or ct scanning or x-ray computed tomography or computer assisted tomography) or (MRI or magnetic resonance imaging or mr imaging or nuclear magnetic resonance imaging or nmr imaging) or (ultrasonography or ultrasonography or echography or ultrasonogram or ultrasonic scanning or ultrasound scanning or ultrasound scan)).mp.	5,174,558
2. deep learning/ or deep neural network/ or convolutional neural network/ or convolution algorithm/ or (deep learning or hierarchical learning or deep neural network or convolutional neural network or convolution algorithm).mp.	201,686
3. kidney polycystic disease/ or (polycystic kidney or renal polycystic disease or cystic kidney or cystic kidney disease or renal cystic disease or autosomal dominant polycystic kidney or ADPKD).mp.	42,238
4. 1 and 2 and 3	69
5. limit 4 to human	61
6. limit 5 to humans	61
7. limit 6 to english language	61
8. limit 6 to danish language	0
9. limit 7 to yr=“2000 -Current”	61

## Appendix C. Complete Spreadsheet of CLAIM Checklist

A complete version of the CLAIM checklist, including all items and corresponding study evaluations, is available in the following Google Spreadsheet: https://docs.google.com/spreadsheets/d/13IWbrKeBfIYuMSLEU3XMU09bRaJHgochj9pTvI1AxzA/edit?usp=sharing (accessed on 13 November 2025).
